# Serum lipidomic study of long-chain fatty acids in psoriasis patients prior to and after anti-IL-17A monoclonal antibody treatment by quantitative GC‒MS analysis with in situ extraction

**DOI:** 10.1186/s12944-023-01999-6

**Published:** 2024-01-08

**Authors:** XiaoYu Guo, Jianglu Zhou, Hong Yu, Han Cao, Xia Li, Qing Hu, YunQiu Yu

**Affiliations:** 1 School of Pharmacy, Fudan University, Shanghai, 201203 PR China; 2 NMPA Key Laboratory for Quality, Control of Traditional Chinese Medicine, Shanghai Institute for Food and Drug Control, Shanghai, 201203 PR China; 3 Department of Dermatology, Ruijin Hospital, School of Medicine, Shanghai Jiaotong University, Shanghai, 200025 PR China

**Keywords:** Long-chain fatty acids, Gas chromatography‒mass spectrometry, Psoriasis, Targeted metabolomics

## Abstract

**Background:**

Long-chain fatty acids (LCFAs) are involved in regulating multiple physiological processes as signalling molecules. Gas chromatography–mass spectrometry (GC–MS) is widely used to quantify LCFAs. However, current quantitative methods for LCFAs using GC–MS have demonstrated complicated issues. Psoriasis is a chronic inflammatory skin disease, and its pathogenesis may be related to the overproduction of interleukin-17A (IL-17A). Clinical efficacy of anti-IL-17A monoclonal antibody (mAb) treatment in psoriasis patients has been demonstrated. Recent studies suggest that LCFAs play varying roles in the pathogenesis of psoriasis. However, more comprehensive research is needed to illuminate the mechanism of LCFAs in psoriasis.

**Methods:**

The established in situ derivatization method for analysing LCFAs with a GC–MS platform was utilized to conduct serum lipidomics analysis of healthy volunteers and psoriasis patients receiving pretherapy and posttreatment with of anti-IL-17A mAb. Imiquimod (IMQ)-treated wild type (WT) and T-cell receptor delta chain knock-out (*Tcrd*^*−/−*^) mice were used to investigate the correlation between IL-17A and abnormal changes in LCFAs in psoriasis patients.

**Results:**

A rapid and sensitive in situ extraction derivatization method for quantifying LCFAs using GC–MS was established. Serum lipidomic results showed that psoriasis patients had higher levels of saturated fatty acids (SFAs) and ω-6 polyunsaturated fatty acids (PUFAs) but lower levels of monounsaturated fatty acids (MUFAs) and ω-3 PUFAs than healthy individuals, indicating impaired serum LCFA metabolism. Anti-IL-17A mAb treatment affected most of these LCFA changes. Analysis of LCFAs in IMQ-treated mice showed that LCFAs increased in the serum of WT mice, while there were no significant changes in the *Tcrd*^*−/−*^ mice. SFAs increased in IMQ-treated WT mice, while MUFAs showed the opposite trend, and PUFAs did not change significantly.

**Conclusions:**

This study presented a dependable method for quantifying LCFAs that enhanced sensitivity and reduced analysis time. The lipidomic analysis results showed that anti-IL-17A mAb not only ameliorated skin lesions in psoriasis patients but also affected abnormal LCFAs metabolism. Furthermore, the study indicated a potential correlation between IL-17A and abnormal LCFA metabolism in psoriasis patients, which was supported by the alterations in serum LCFAs observed in IMQ-treated WT and *Tcrd*^*−/−*^ mice.

**Supplementary Information:**

The online version contains supplementary material available at 10.1186/s12944-023-01999-6.

## Introduction

Fatty acids (FAs) belong to the lipid family and are structurally simple carboxylic acids, that are not only used to form other complex lipids but also act as signalling molecules to regulate multiple physiological processes [1, 2]. Long-chain fatty acids (LCFAs) are FAs chains with 12-26 carbons and can be categorized into saturated fatty acids (SFAs), monounsaturated fatty acids (MUFAs) and polyunsaturated fatty acids (PUFAs) [3].

Various methods have been developed to quantify LCFAs and investigate their role in disease pathogenesis [4, 5]. These methods have allowed researchers to investigate the relationship between LCFAs and disease development in a variety of biological samples. Gas chromatography–mass spectrometry (GC–MS) has become a widely used analytical tool for LCFA quantification due to its high selectivity, low solvent consumption, and efficient analysis [6, 7]. However, current quantitative methods for LCFAs in serum samples using GC–MS analysis platforms have demonstrated issues with complicated preprocessing procedures, long analysis cycle durations, and low method sensitivity [8]. Lipidomic research often involves analysing and processing large amounts of biological samples quickly. To meet these demands, it is important to develop a GC–MS based method for quantifying LCFAs in biological samples that is simple, efficient, and highly sensitive and has a short preprocessing time.

Psoriasis is a systemic skin inflammatory disease caused by multiple factors, with interleukin (IL)-23/17A playing a crucial role in its pathogenesis [9, 10]. Use of an anti-IL-17A monoclonal antibody (mAb) has shown effectiveness in treating severe plaque psoriasis [11]. However, the drawbacks of the treatment, including high cost and frequent recurrence, should not be ignored. Recent studies have suggested that LCFA are “double-edged swords” that may play anti-inflammatory or proinflammatory roles in the pathogenesis of psoriasis [12]. MUFAs in the skin can inhibit ultraviolet radiation-induced skin inflammation by suppressing cyclooxygenase-2 [13]. Treg cells stimulated by omega-3 (ω-3) PUFAs generate more anti-inflammatory factors, thereby providing protection against imiquimod (IMQ)-induced psoriasis in mice [14]. In contrast to MUFAs and ω-3 PUFAs, SFAs activate immune cells to release more proinflammatory factors [15]. Furthermore, ω-6 PUFAs generate leukotriene metabolites under the action of lipoxygenase, promoting inflammatory skin reactions in psoriasis patients [16]. However, studies of LCFAs and their effects on psoriasis have reported conflicting conclusions. A targeted lipidomic study of healthy participants and psoriasis patients revealed that as the disease progresses, psoriasis patients experience a significant increase in PUFAs, MUFAs and SFAs [17]. Conversely, another quantitative analysis study of LCFAs found that psoriasis patients had greater levels of MUFAs and reduced levels of SFAs and PUFAs in serum samples compared to healthy individuals [18]. While these studies had varying conclusions, they both suggested that psoriasis patients had metabolic abnormalities in LCFAs, which could impact the pathogenesis of the condition.

Previous research has shown that psoriasis patients have an abnormal metabolic profile of LCFAs. However, the correlation between the metabolic profile of LCFAs and the key pathogenic factor IL-17A has not yet been established. In this study, a simple, accurate, and sensitive method was developed to quantitatively analyse endogenous LCFAs in serum samples. Using this method, this study compared the differences in LCFA metabolism between healthy individuals and psoriasis patients and examined the changes in LCFAs in each psoriasis patient receiving pretherapy and posttreatment with anti-IL-17A mAb. The correlation between the key pathogenic factor IL-17A and abnormal LCFA metabolism in psoriasis patients was further supported using IMQ-treated wild-type (WT) and T-cell receptor delta chain knockout (*Tcrd*^*−/−*^) mice. To our knowledge, this study is the first to utilize a validated biological analysis method to compare the differences in LCFA metabolism in psoriasis patients receiving pretherapy and posttreatment with anti-IL-17A mAb. These findings not only reveal the correlation between abnormal LCFA metabolism and IL-17A in psoriasis patients, but also provide a theoretical foundation for the use of anti-IL-17A mAb in treating the skin lesions of psoriasis patients while improving their abnormal LCFA metabolism.

## Methods

### Patients and ethics statement

A total of 75 serum samples were collected from 15 healthy volunteers (HC group), 15 psoriasis patients at onset (PSV group), and 15 psoriasis patients at one, two, and eight weeks after anti-IL-17A mAb (ixekizumab) treatment (W1, W2, W8 groups). The Ethics Committee of Ruijin Hospital of Shanghai Jiao Tong University reviewed and approved the protocol. Written informed consent was obtained from each patient and healthy individuals before enrolment. The severity of psoriasis was quantified by the psoriasis area severity index (PASI) score. These healthy volunteers met the requirements of having no history of obesity-related metabolic disease. Psoriasis patients did not receive any anti-inflammatory therapy for a month. In this study, 15 psoriasis patients received an initial subcutaneous injection of 160 mg of ixekizumab, followed by weekly subcutaneous injections of 80 mg of ixekizumab. All volunteers and patients had the same sex and age (Table 1). Serum samples were collected in ethylenediaminetetraacetic acid tubes, processed according to standard operating procedures (stood at room temperature for four hours and centrifuged at 3000 rpm for five minutes), subpacked and stored in a -80 °C refrigerator.

**Table 1:** Demographic information of different groups

	HC group (*n* = 15)	PSV group (*n* = 15)	W1 group (*n* = 15)	W2 group (*n* = 15)	W8 group (*n* = 15)
Male/Female	11/4	11/4	11/4	11/4	11/4
Age (years)	46 ± 12	47 ± 12	47 ± 12	47 ± 12	47 ± 12
BMI	24.7 ± 2.1	25.3 ± 2.6	28.5 ± 2.8^*#^	29.1 ± 3.7^*#^	26.3 ± 3.4^*#^
PASI	N/A	23.3 ± 10.4	18.1 ± 6.3	13.9 ± 5.4^#^	1.2 ± 1.1^#^

^*^There was a significant difference compared with the HC group, *P < 0.05

^#^There was a significant difference compared with the PSV group, ^#^*P* < 0.05

Values are reported as mean ± SD

### Animals and treatments

*Tcrd*^*−/−*^ transgenic mice were a gift from Professor Jie Zheng, and C57/BL6 wild-type (WT) mice were purchased from Lingchang Biotechnology (Shanghai, China). All animals were bred in the laboratory animal facility of Fudan University School of Pharmacy (Shanghai, China). At 6-7 weeks of age, a daily topical dose of 62.5 mg imiquimod (IMQ) cream (5%, Aldara, MN, USA) or Vaseline cream (Unilever, CT, USA) was applied to the shaved dorsal skin of 24 mice (12 *Tcrd*^*−/−*^ mice and 12 WT mice) for 5 days. Prior to sacrifice, all 24 mice were fed a general diet for 5 days. The Animal Research Ethics Committee of Fudan University School of Pharmacy (Shanghai, China) approved the present study (2021-03-YJ-HRO-07). The experimental procedures strictly adhered to the principles of the National Institutes of Health Guide.

### Chemicals and materials

Lauric acid (12:0), myristic acid (14:0), palmitic acid (16:0), palmitoleic acid (16:1), stearic acid (18:0), arachidonic acid (20:4), eicosapentaenoic acid (20:5), behenic acid (22:0) and tetracosanoic acid (24:0) were purchased from Aladdin Reagent (Shanghai, China). Oleic acid (18:1), linoleic acid (18:2), eicosanoic acid (20:0), docosahexaenoic acid (22:6), hexacosanoic acid (26:0), HPLC-grade pyridine, methoxamine hydrochloride, N-methyl-N-(trimethylsilyl) trifluoroacetamide, and BF_3_-CH_3_OH solution were purchased from Sigma–Aldrich (St. Louis, MO, USA).

2-d_2_-Lauric acid (12:0), 2,2-d_2_-myristic acid (14:0), 1,2-^13^C_2_-palmitic acid (16:0), U-^13^C_16_-palmitoleic acid (16:1), 2,2-d_2_-stearic acid (18:0), 9,10-d_2_-oleic acid (18:1), 1-^13^C-linoleic acid (18:2), 2,2-d_2_-eicosanoic acid (20:0), 5,6,8,9,11,12,14,15-d_8_-arachidonic acid (20:4), 19,19,20,20,20-d_5_-cis-eicosapentaenoic acid (20:5), 12,12,13,13-d_4_-behenic acid (22:0), U-^13^C_22_-docosahexaenoic acid (22:6), 12,12,13,13-d_4_-etracosanoic acid (24:0) and 12,12,13,13-d_4_-hexacosanoic acid (26:0) were purchased from Cambridge Isotope Laboratories (Xenia, OH, USA.).

For the targeted analysis of LCFAs, the standard solutions and isotope standard solutions were separately stocked in methanol (Sigma–Aldrich, St. Louis, MO, USA) at a concentration of 2 mg/mL. Subsequently, the working mixed standards were processed by mixing the stock solutions and performing a serial dilution in methanol. The storage solutions were kept at -20 °C, while the working solutions were stored at -4 °C.

### Scoring severity of skin inflammation and histology

The PASI score was objectively used to evaluate the severity of inflammation, including erythema, scale and skin thickening, in psoriasis patients. The scoring criteria were 0 to 4, with 0 indicating no symptoms and 4 indicating highly marked symptoms. The total score was obtained by adding the three subscale scores. The severity of the IMQ-treated mice could be measured with the PASI score. This scoring system takes into account the fixed area with IMQ treatment and excludes the affected skin area.

The dorsal skin tissues of mice were fixed in formalin and embedded in paraffin, followed by staining with H&E. ImagePro Plus software (Leeds Precision Instruments, MN, USA) was used to measure the epidermal thickness, and the data obtained from a series of rectangles were divided by the total length of the epidermis to calculate the total epidermal area.

### Preparation of serum samples by in-situ and two-way extraction

Thirty microlitre freeze–thawed serum samples were combined with 500 μL NaOH-methanol solution. To separate LCFAs from serum proteins, the mixture was vortexed for 60 s and allowed to stand on ice for 15 min. Next, 500 μL of n-hexane was added to the mixture and vortexed it for 3 min. The methanol layer was separated by centrifugation at 12000 rpm for 5 min. A total of 450 μL of methanol layer solution was removed, dried with nitrogen at 40 °C and then redissolved in 30 μL of methanol. For the two-way extraction, the reconstituted sample was combined with 30 μL of isotope standard solution. Then, the mixture was vortexed for 30 s and heated over an iron bath at 50 °C for 30 min. After cooling, 1 mL of dichloromethane was added, and the mixture was centrifuged at 12,000 rpm for 5 min to obtain fatty acid methyl esters (FAMEs). This step was repeated three times. For the in suit extraction, the reconstituted sample was combined with 3 0μL of isotope standard solution, 500 μL of 15% BF_3_-CH_3_OH solution, and 1 mL of dichloromethane extraction solvent. After vortexing for 30 s, the mixture was sealed and heated at 40 °C for 30 min. It was then centrifuged at 12,000 rpm for 10 min. The dichloromethane phase obtained through the two derivatization methods was evaporated to dryness under nitrogen flow and reconstituted in 200 μL of n-hexane, followed by vortexing and centrifugation at 12,000 rpm for 5 min. Finally, the supernatant was injected into a 7890B gas chromatograph coupled to a 5977A quadrupole mass spectrometer (Agilent Technologies, Santa Clara, CA, USA) for analysis. Chromatographic separations of LCFAs were performed using an HP-5 MS capillary column (30 m × 250 μm × 0.25 μm).

### Isotope dilution quantitative method

The LCFA standard and isotope standard solutions were prepared with different concentration gradients and subjected to linear regression analysis. The response factor (R_f_) was calculated using the following formula: R_f_ = K_RS_/ K_ISO_, where K_RS_ represents the linear slope of the standard solution and K_ISO_ is the linear slope of the isotope standard solution. The stability of the R_f_ values was measured every ten days, and the relative standard deviation (RSD)% was calculated after repeating the measurement three times to ensure reproducibility. In the quantitative sample analysis, the target LCFA concentration was calculated using the following formula: C_x_ = (A_x_ × C_ISO_)/(A_ISO_ × R_f_), where A_x_ and A_ISO_ represented the peak areas of the targeted LCFAs and isotope standards, respectively, C_ISO_ represented the concentration of the isotope standards added, and C_x_ represented the final quantitative concentrations of the targeted LCFAs.

### Method validation

The method involved standard validation procedures, such as linear analysis of calibration curves, limit of detection (LOD), limit of quantification (LOQ), precision and recovery. To evaluate the linearity of the standard curves, five concentration points were tested and each point was repeated thrice. The LCFA concentrations were used as the LOD when the signal-to-noise ratio (S/N) was greater than or equal to 3 and as the LOQ when S/N was greater than or equal to 10. The Agilent MassHunter Quantitative Analysis workstation (Agilent Technologies, Santa Clara, CA, USA) was utilized to calculate the S/N ratio. Intraday precision was assessed with five repeated analyses of low, medium and high concentrations in each LCFA linear range on the same day. The interday precision assessment method was repeated analysis of low, medium and high concentrations in each LCFA linear range for three consecutive days. This approach ensured that the concentration of the actual samples tested fell within the range of the lowest and highest concentrations and approached the average concentration [19]. By doing so, the experimental accuracy was enhanced. Recovery was calculated using the following formula: (A_1_-A_2_)/A_x_, where A_1_ represented the concentration of analytes in human serum with added standard samples, A_2_ represented the concentration of analytes in human serum without added standard samples, and A_x_ represented the concentration of the added standard samples. A recovery experiment was conducted to evaluate the systematic error in the quantitative process and validate the accuracy of the analytical method [20]. The recovery results were calculated at three different concentrations (low, medium, and high) to assess the suitability of the method for various concentrations of LCFAs.

### Statistics

Prior to comparing means between different groups, the raw data were logarithmically transformed and tested for normality. Student's t test was used if normality was assumed, or a non-parametric method was applied if normality was not assumed. The mean values between the four groups were compared using analysis of variance and then compared with the control group using the Dunnett's t test. All statistical analyses were performed using SPSS v20 (IBM, IL, USA). Linear regression models and prediction plots were generated using Prism 8.0 (GraphPad Software Inc., San Diego, CA, USA).

Model diagnostics for linear mixed models play a crucial role in evaluating the agreement between the model and the data. This assessment was conducted through marginal and conditional residual plots, which test for normality, linearity, homogeneity of the residuals, and anomalies. Additionally, the evaluation of influential observations is an important diagnostic measure that helps identify extreme or abnormal values [21]. The analyses of linear mixed models were performed using a combination of the SAS system (version 9.4) and R software (version R4.1.2).

## Results

### GC–MS condition optimization

**Fig. 1:**
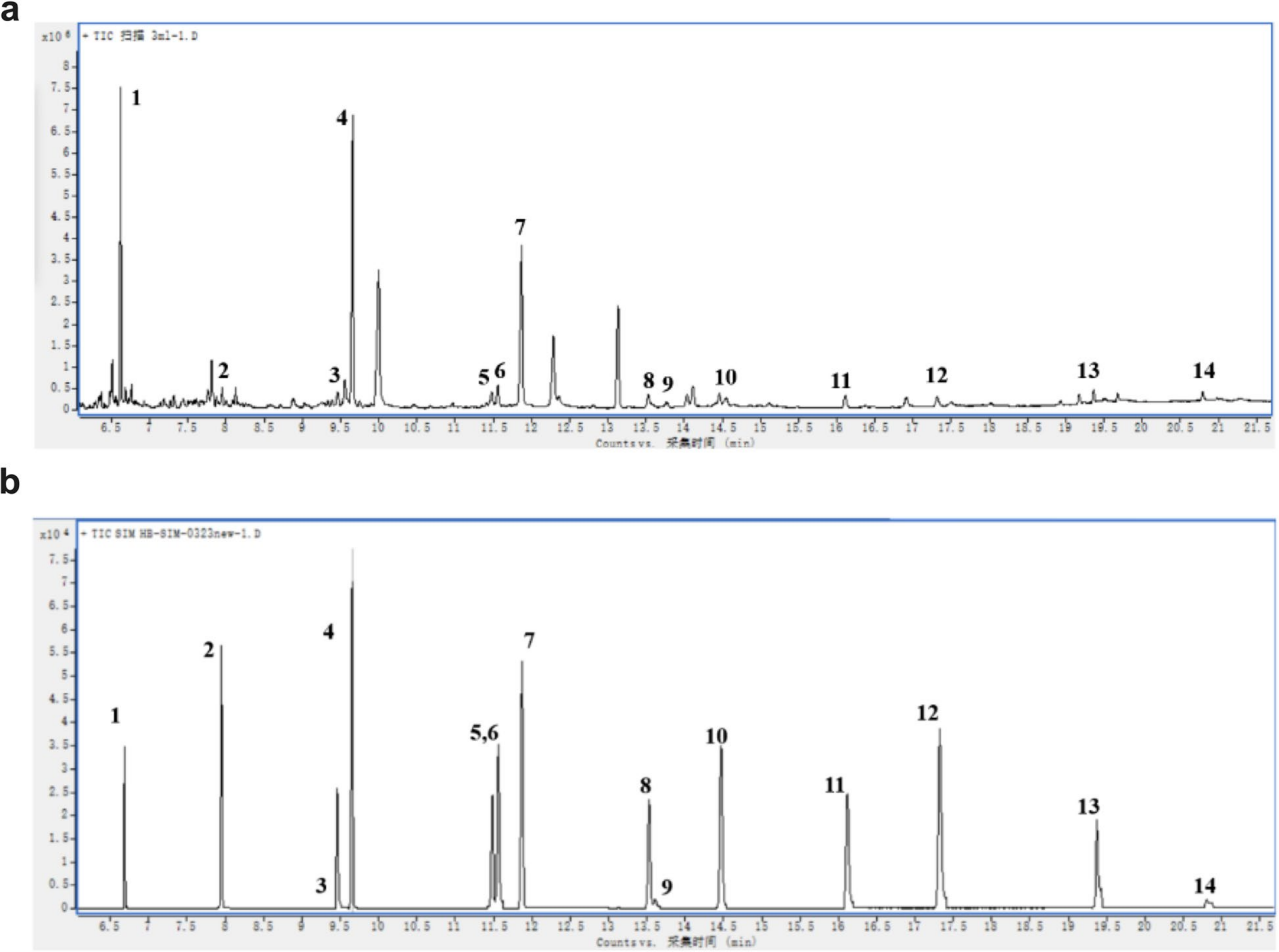
Chromatogram of 14 LCFA mixed standard solutions by in situ extraction method in full-scan mode (**a**) and SIM mode (**b**). 1: lauric acid (12:0); 2: myristic acid (14:0); 3: palmitoleic acid (16:1); 4: palmitic acid (16:0); 5: linoleic acid (18:2); 6: oleic acid (18:1); 7: stearic acid (18:0); 8: arachidonic acid (20:4); 9: eicosapentaenoic acid (20:5); 10: eicosanoic acid (20:0); 11: docosahexaenoic acid (22:6); 12: behenic acid (22:0); 13: tetracosanoic acid (24:0); 14: hexacosanoic acid (26:0)

After optimizing the GC temperature program, all 14 LCFAs were successfully separated within a 21.7-min analysis cycle, indicating that the method had high analytical efficiency and a short analysis time (Fig. 1a). In comparison to the full-scan mode, the selected ion monitor (SIM) mode had better sensitivity and resolution as well as multiple ion detection advantages in GC–MS analysis. Therefore, the SIM mode was more advantageous for the quantification of 14 LCFAs (Fig. 1b). The retention time, qualitative ions, and quantitative ions for the 14 LCFAs in SIM mode are shown in Table S1.

### LCFA derivatization method optimization

To analyse LCFAs with GC–MS, it is necessary to gasify them through derivatization prior to analysis [22]. The most commonly used derivative products for LCFAs are FAMEs [23]. A comparison of common acid derivatization agents revealed that BF_3_ had higher efficiency than HCl and H_2_SO_4_ for most LCFAs (Fig. S1a). Further investigation into the concentration and quantity of the derivatization agent revealed that LCFAs had the optimal derivatization efficiency in a 15% BF_3_-CH_3_OH solution (Fig. S1b). Using too little of the derivatization agent results in an incomplete reaction, while using too much excessively dilutes the target analytes, and the optimal reaction concentrations cannot be achieved. In the study comparing different amounts of derivatization agents, it was discovered that adding 500 μL BF_3_-CH_3_OH solution yielded better derivatization efficiency (Fig. S1c). The temperature and time of the derivatization reaction were also analysed, and it was found that LCFAs had the highest derivatization efficiency at 40 °C for 30 min (Fig. S1d, e). The extraction efficiency of n-hexane, dichloromethane, chloroform, and ethyl acetate as extracting solvents for FAMEs was evaluated. The results indicated that dichloromethane had the best extraction efficiency, which may be attributed to its lower boiling point, higher relative density, and location below the methanol layer, allowing for increased interfacial contact between the two phases after reaching the boiling point (Fig. S1f).

### Method validation

The calibration curves for LCFAs and isotope standard solutions were in terms of concentration and response and a showed significant correlation (R^2^ ≥ 0.9990) even without the use of any weighting techniques. Additionally, all calibration curves demonstrated reproducible outcomes. The results of the LOD and LOQ suggested that SFAs had a higher detection sensitivity than MUFAs and PUFAs, with the detection sensitivity of the latter two groups being relatively low. The analytical method exhibited high sensitivity and met the quantitative analysis requirements, as evidenced by the LOD values ranging from 1.01-4999.82 nM and LOQ values ranging from 5.01-9999.97 nM across the 14 LCFAs (Table 2). Compared to previous studies that used the GC–MS analysis platform to measure serum LCFAs (with LOD values ranging from 0.02-17 μM and 0.6-50 μM), the established quantification method in this study demonstrated higher sensitivity [24, 25].

**Table 2:** Linear range, regression coefficient, and calibration equation of LCFAs

LCFA	Range (μM)	*R*^2^	ISO-range (μM)	ISO-R^2^	R_f_ value	RSD of R_f_ (%)	LOD (nM)	LOQ (nM)
12:0	0.05-40.01	0.9996	0.05-40.41	0.9997	1.33	9.82	5.01	25.01
14:0	0.51-199.98	0.9993	0.51-201.78	0.9996	1.18	15.29	1.02	5.01
16:0	25.01-14998.59	0.9990	25.19-15115.58	0.9993	0.58	9.01	1.01	5.01
16:1	1.01-199.99	0.9994	1.06-212.57	0.9994	1.21	1.21	49.99	100.01
18:0	10.01-4000.32	0.9993	10.07-4028.44	0.9994	1.20	2.27	1.01	5.02
18:1	25.01-7498.37	0.9991	25.18-7555.01	0.9991	0.89	2.88	1.01	5.01
18:2	25.01-3000.23	0.9993	25.09-3010.92	0.9997	1.31	7.33	1.01	5.01
20:0	0.05-50.01	0.9997	0.05-50.33	0.9999	2.02	11.73	5.02	49.98
20:4	0.51-400.04	0.9990	0.51-410.55	0.9994	10.42	11.14	5.01	50.02
20:5	10.01-499.92	0.9996	10.16-508.18	0.9992	1.08	15.14	4999.82	9999.97
22:0	0.05-50.01	0.9998	0.05-50.59	0.9997	1.42	7.57	25.01	33.33
22:6	3.25-499.87	0.9993	3.56-533.35	0.9993	3.16	8.34	1000.01	2499.99
24:0	0.51-80.01	0.9991	0.05-80.87	0.9993	0.93	1.01	5.01	500.01
26:0	0.25-80.01	0.9990	0.25-80.82	0.9993	3.66	17.56	25.01	49.99

*ISO* isotope standard solutions

During the development of the method, it was observed that the instrument response values of the standard solutions and isotope standard solutions with the same concentration were not the same. To account for this difference caused by the purity or derivatization efficiency and ionization efficiency of isotope standards, the coefficient R_f_ value was introduced in isotope dilution mass spectrometry [26]. The Rf value was defined as the ratio between the slope of the calibration curve of each LCFA and the slope of the calibration curve of its isotope. The Rf values corresponding to LCFAs, ranging from 0.58 to 10.4, indicated that the instrument response values of most LCFA isotope standards were similar to those of the standards. In this study, the Rf values were calculated linearly through repeated measurements every ten days. The RSD (%) of Rf values for LCFAs in the three replicates ranged from 1.01% to 17.56%, suggesting that the Rf values were generally reproducible and could serve as a stable correction coefficient for quantitation. These R_f_ values can be utilized as a reliable correction coefficient for quantitative analysis (Table 2).

The study evaluated the recovery rates and precision of 14 LCFAs at high, medium, and low concentrations. The results showed that the recovery rates of these LCFAs in the serum matrix ranged between 92 and 106%. There was no significant signal enhancement or inhibition, and the matrix effect was relatively small. The RSD% of all LCFAs at low, high and medium concentrations was within 15 (Table 3).

**Table 3:** Recovery, Intraday and Interday precision of LCFAs

LCFAs	Recovery (%)			Intraday precision (%) (*n* = 5)			Interday precision (%) (*n* = 5)		
	L	M	H	L	M	H	L	M	H
12:0	96.31	94.41	95.90	0.05	0.57	2.78	0.07	0.46	4.67
14:0	98.81	97.94	98.43	0.16	1.77	3.73	0.31	2.03	9.89
16:0	98.33	97.79	97.50	0.70	2.39	7.76	0.42	4.85	9.11
16:1	102.67	95.99	101.00	0.13	3.65	2.84	0.20	2.11	4.16
18:0	104.77	97.41	92.36	0.34	2.32	6.35	0.21	1.68	9.31
18:1	101.80	97.86	98.01	1.44	5.61	8.86	2.24	9.98	14.90
18:2	98.78	98.16	98.76	0.09	3.19	7.75	0.31	4.16	7.91
20:0	95.62	94.86	93.58	1.12	0.46	14.94	3.20	4.04	14.43
20:4	92.68	102.81	93.19	0.08	0.18	12.90	0.05	0.59	14.19
20:5	103.57	105.36	92.70	0.53	0.41	11.18	0.47	8.07	14.03
22:0	97.61	103.37	96.44	0.30	3.63	8.15	1.28	4.61	14.72
22:6	101.76	96.84	98.42	0.32	1.02	5.45	0.97	1.57	14.33
24:0	105.15	96.61	93.50	2.79	5.34	8.93	1.81	5.59	13.32
26:0	94.76	92.96	105.41	1.47	4.19	8.71	1.48	6.26	14.31

*L* Low concentration, *M* Medium concentration, *H* High concentration

### Comparison of two quantitative methods

**Fig. 2:**
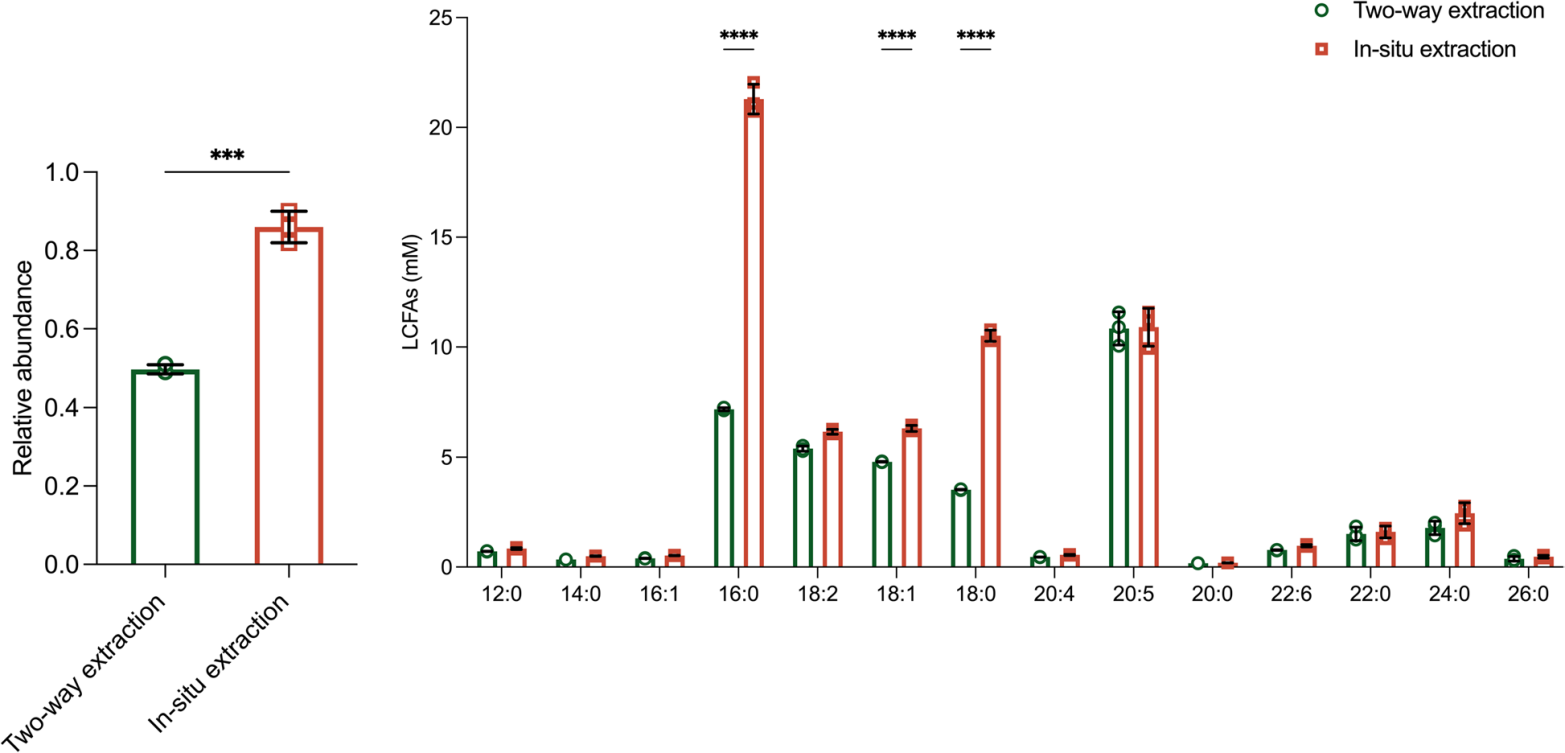
Relative abundance of 14 LCFAs by two-way and in situ extraction methods. *P < 0.05, **P < 0.01, ***P < 0.001, ****P < 0.0001

An in situ extraction method was developed to simultaneously derive LCFAs and extract FAMEs. This method eliminated the need for solvents to methylate LCFAs into FAMEs and instead the extraction solvent is directly added during the derivation process. This method enhanced the extraction efficiency and reduced the preprocessing time. Compared to the traditional two-way method, the in situ extraction method demonstrated a significant improvement in the signal response for palmitic and stearic acid in the GC–MS system, with 33.1% and 33.2% increases, respectively (Fig. 2). Additionally, LCFA in situ extraction increased the response of myristic acid, palmitoleic acid, oleic acid, docosahexaenoic acid, tetracosanoic acid, and hexacosanoic acid by 15.6%, 11.6%, 12.1%, 10.1%, 13.4%, and 9.9%, respectively. Overall, the in situ extraction method simplified the preprocessing step, reduced the analysis time, and improved LCFA derivatization and FAME extraction efficiency. This methodology was particularly important for high-throughput sample preparation in lipidomic studies.

### Quantification of LCFAs in the serum of psoriasis patients receiving pretherapy and posttreatment with anti-IL-17A mAb

**Fig. 3:**
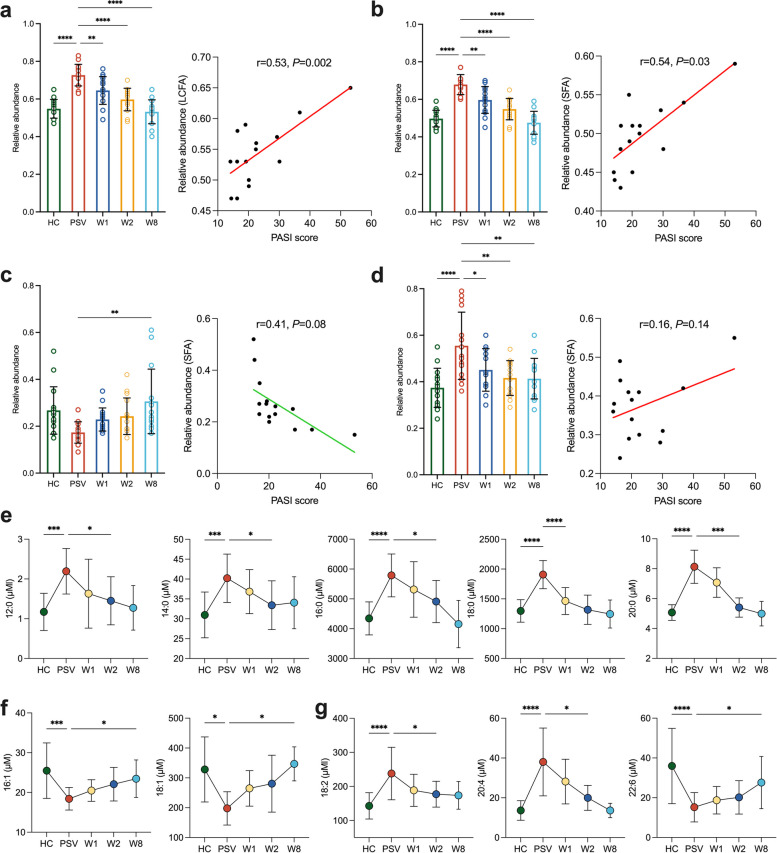
Anti-IL-17A mAb regulated abnormal LCFA metabolism in psoriasis patients. The total LCFA (**a**), SFAs (**b**, **e**), MUFA (**c**, **f**) and PUFA (**d**, **g**) contents in the serum samples of healthy individuals and psoriasis patients receiving pretherapy and posttreatment with anti-IL-17A mAb. *P < 0.05, **P < 0.01, ***P < 0.001, ****P < 0.0001

To investigate the correlation of LCFAs in the pathogenesis of psoriasis, the established in situ extraction method was used to analyse LCFAs in the serum samples of healthy volunteers and psoriasis patients receiving pretherapy and posttreatment with anti-IL-17A mAb. Ten different LCFAs were detected during the quantification of serum samples (Table S2). The LCFA content in psoriasis patients was significantly higher than that in healthy individuals (Fig. 3a). During extended treatment with anti-IL-17A mAb, LCFAs in psoriasis patients gradually decreased, consistent with changes in the PASI scores before and after treatment. Linear analysis showed a strong positive correlation between LCFAs and the PASI score in psoriasis patients (Fig. 3a).

This study found that the content of LCFAs in the serum samples of healthy volunteers was lower than that of psoriasis patients and that the trends of different types of LCFAs were not the same. SFAs, such as palmitic acid (5787.41 ± 718.52 μM) and stearic acid (1906.71 ± 235.23 μM), were upregulated in psoriasis patients and decreased significantly after anti-IL-17A mAb treatment (Fig. 3b). This significant difference was observed after just one week of treatment (Fig. 3e). On the other hand, MUFAs, represented by palmitoleic acid (18.42 ± 2.84 μM), were downregulated in psoriasis patients but showed a significant increase after anti-IL-17A mAb treatment (22.14 ± 4.79 μM) (Fig. 3c). The increase in MUFAs after treatment did not show significant differences until the eighth week, which was inconsistent with the changes observed in SFAs (Fig. 3f). This study also analysed the correlations between SFAs, MUFAs, and the PASI scores in psoriasis patients and found that with the increasing PASI scores, the SFA content increased considerably, while MUFAs showed a negative correlation (Fig. 3b, c).

This study found a significant reduction in ω-3 PUFAs, specifically docosahexaenoic acid (15.21 ± 7.13 μM), in the serum of psoriasis patients. Conversely, ω-6 PUFAs, represented by arachidonic acid (38.02 ± 17.07 μM), showed a marked increase (Fig. 3g). A targeted metabolomic study comparing healthy individuals and patients with autoimmune diseases, including psoriasis, found a decrease in serum docosahexaenoic acid (105.7 ± 50.9 μM) and an increase in arachidonic acid (424.4 ± 124.7 μM) among patients with autoimmune diseases, supporting the findings of this study [27]. After receiving anti-IL-17A mAb treatment, there was a decreasing trend in ω-6 PUFAs and a significant increase in ω-3 PUFAs, consistent with MUFAs, with significant differences detected in the second and eighth weeks of treatment (Fig. 3g). However, although there were no strong correlations between the PUFA and ω-6 PUFA content and PASI scores in psoriasis patients, there was a weak positive correlation between the ω-3 PUFAs and PASI scores (Fig. S2).

Receiver operating characteristic (ROC) curve analysis is widely accepted as one of the reliable criteria for evaluating biomarker function. The ROC curves of the 10 LCFAs in the PSV/HC comparison had area under the curve (AUC) values greater than 0.8, suggesting that the LCFAs had the potential to be biomarkers for psoriasis (Fig. S3a). Notably, arachidonic acid and eicosanoic acid had AUC values close to 1.0 in both the PSV/HC comparison and W8/PSV comparison, indicating that they can be used as predictive markers to evaluate the efficacy of anti-IL-17A mAb therapy in psoriasis patients (Fig. S3b).

### Quantification of LCFAs in the serum of IMQ-treated WT and *Tcrd*^−^/^−^ mice

**Fig. 4:**
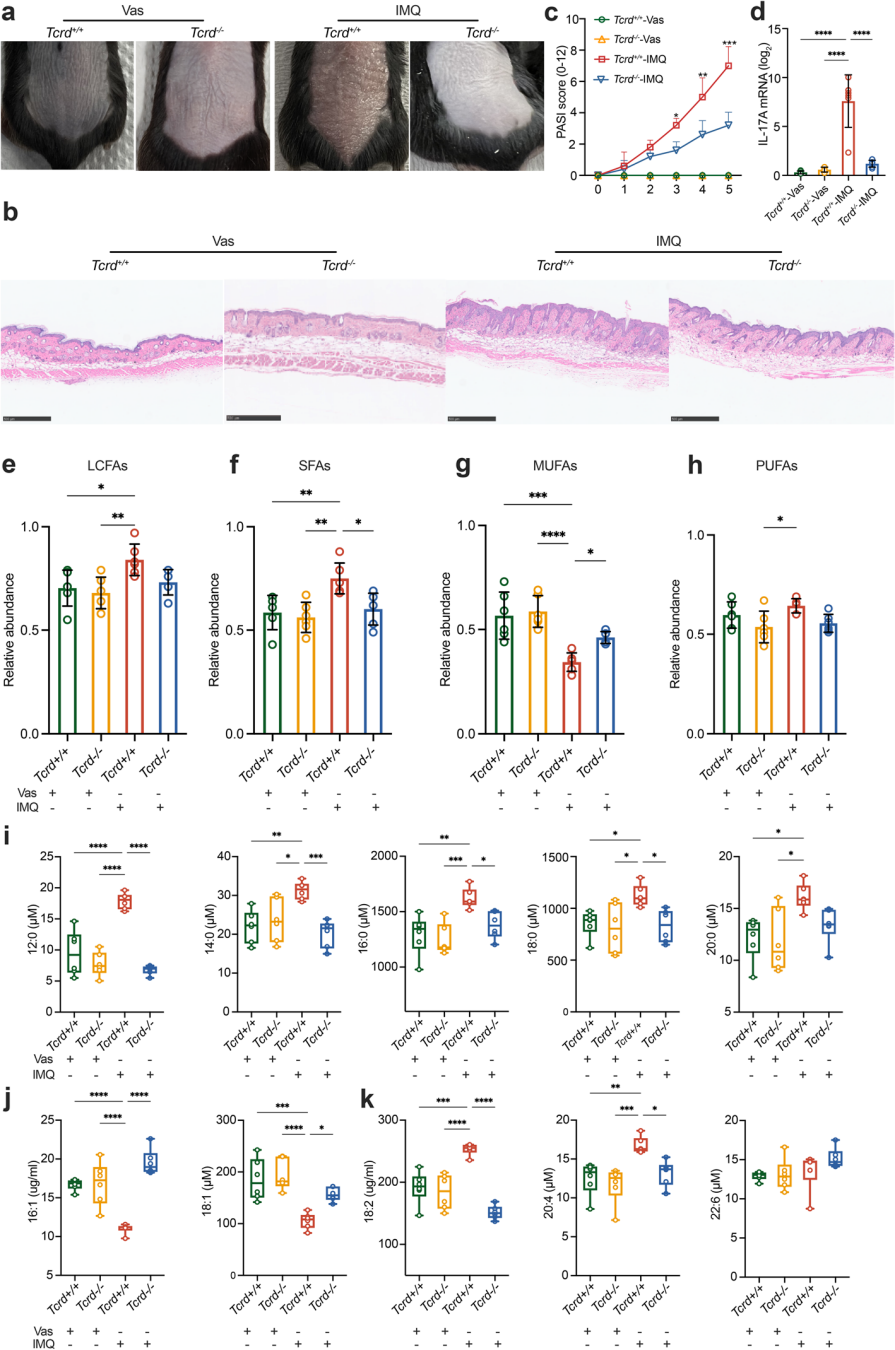
Abnormal LCFA metabolism in IMQ-treated WT and *Tcrd*^*−/−*^ mice. Macroscopic and histological views of the dorsal skin were taken after continuous treatment with IMQ for five days (**a**, **b**). PASI scores were identified on the days indicated on a scale from 0–12 (**c**). IL-17A mRNA expression in the dorsal skin of IMQ-treated WT and *Tcrd*^*−/−*^ mice was measured (**d**). The total content of LCFAs (**e**), SFAs (**f**, **i**), MUFAs (**g**, **j**) and PUFAs (**h**, **k**) in serum samples of WT and *Tcrd*^*−/−*^ mice was analysed. *P < 0.05, **P < 0.01, ***P < 0.001, ****P < 0.0001

To investigate the relationship between IL-17A and abnormal changes in LCFAs in psoriasis patients, IMQ-treated WT and *Tcrd*^*−/−*^ mice were used to conduct experiments. Previous studies have demonstrated that γδ T cells are the main producers of IL-17A in the mouse dermis [28, 29]. Due to the absence of γδ T cells, the IL-17A content was significantly reduced in the skin of *Tcrd*^*−/−*^ mice. Both WT and *Tcrd*^*−/−*^ mice were subjected to IMQ treatment under identical conditions. After applying IMQ cream for 5 days, the dorsal skin lesions of the WT mice increased significantly, while the skin lesions of the *Tcrd*^*−/−*^ mice did not show significant changes. Compared to IMQ-treated *Tcrd*^*−/−*^ mice, IMQ-treated WT mice had more scales, redness and a thicker skin epidermis than IMQ-treated *Tcrd*^*−/−*^ mice (Fig. 4a). The increase in skin epidermis was more obvious in the IMQ-treated WT mice, as confirmed by the H&E staining results (Fig. 4b). There were also significant differences in the PASI scores between the IMQ-treated WT and *Tcrd*^*−/−*^ mice on the third day (Fig. 4c). Although the RT–PCR results revealed that the content of IL-17A in IMQ-treated mice was increased comparing with that in the control group, the expression of IL-17A in IMQ-treated *Tcrd*^*−/−*^ mice was significantly lower than that in WT mice (Fig. 4d).

Similar to the analysis results of LCFAs in the serum of psoriasis patients before and after anti-IL-17A mAb treatment, a total of 10 LCFAs were accurately detected in the serum of psoriasis-like mice (Table S3). The SFAs in the serum of WT mice treated with IMQ were significantly increased compared to those in the control group, while MUFAs exhibited the opposite trend (Fig. 4e-g). There were no significant changes observed in PUMAs (Fig. 4h). However, there was no change in SFAs, MUFAs and PUFAs in the serum of *Tcrd*^*−/−*^ mice, regardless of IMQ exposure (Fig. 4i-k).

## Discussion

This study presented an in-situ derivatization method for quantifying LCFAs in serum samples using a high-throughput GC–MS platform. The method involved using the extraction solvent during the entire reaction, which enhanced the extraction efficiency of FAMEs, the derivatization products of LCFAs. By simultaneously deriving LCFAs and extracting FAMEs, the preprocessing time was reduced. The efficiency of derivatization is affected by diverse determinants, including the concentration, quantity, reaction temperature, and type and time of the derivatization agents. Compared to HCl and H_2_SO_4_, BF_3_ had a higher derivatization efficiency for most LCFAs. To achieve optimal efficiency, LCFAs should be heated in a 15% BF_3_-CH_3_OH solution at 40 °C for 30 min. Dichloromethane is also recommended as a superior extraction solvent.

This study utilized the isotope dilution quantification method to enhance the accuracy of quantification. Isotope dilution mass spectrometry, a technique where a known concentration of stable isotopes with determined mass and abundance is introduced to the sample as an internal standard, was employed [30]. The concentration of the target analyte was measured by the peak area ratio between the target analyte and the isotope standard. As the stable isotope-labelled internal standards and the analytes have identical structures, they exhibit highly similar chromatographic retention times and ionization efficiencies. This study utilized isotope dilution mass spectrometry to accurately quantify LCFAs in serum samples. The correction factor R_f_ was calculated to ensure that response differences were rectified, resulting in greater accuracy of the quantification method [26]. The stability of the R_f_ value was examined to ensure reliability for long-term measurements. Method validation results showed excellent linearity (R2 > 0.9990), recovery rates (92%-106%), and precision (intraday and interday RSD < 15%), making it a suitable method for serum lipidomic analysis.

Psoriasis is a systemic disease induced by various factors, in which the IL-17A pathogenic axis playing a crucial role [31]. This study utilized the established in-situ derivatization method to conduct a targeted lipidomic analysis of LCFAs in serum samples from healthy volunteers and psoriasis patients receiving pretherapy and posttreatment with anti-IL-17A mAb. Considering the potential influence of body mass index (BMI) on the quantitative results of LCFAs in serum samples among psoriasis patients, although healthy volunteers and psoriasis patients had similar BMIs, a significant increase in BMI was observed after anti-IL-17A mAb treatment. To evaluate the influence of BMI on alterations in LCFAs in the serum of the PSV group and W1-8 groups, a linear mixed effects model was employed. Each model incorporated the expression levels of LCFAs as the dependent variables, BMI and anti-IL-17A mAb treatment as independent variables, and patient identities as random effects. The results of the analysis using the linear mixed effects model indicated that BMI did not exert a confounding effect on the observed changes in LCFAs after anti-IL-17A mAb treatment (Table 4). In psoriasis patients, both BMI and weight increased significantly with the use of anti-TNF-α therapies [32, 33]. However, conflicting evidence exists regarding BMI changes in patients with psoriasis receiving anti-IL-17A mAb therapy. A preliminary comparative study suggested that the use of ixekizumab in psoriasis patients did not affect body weight and proposed it as a more favourable decision for overweight patients receiving anti-TNF-alpha therapy [34]. On the other hand, an exploratory post hoc analysis revealed that psoriasis patients experienced weight loss after 52 weeks of IL-17A treatment [35]. Additionally, a retrospective study reported significant increases in BMI and weight in patients with psoriasis after 24 weeks of anti-IL-17A mAb treatment [36]. It’s had been shown that IL-17A inhibites adipocyte differentiation by influencing the combined action of multiple transcription factors, thereby impeding adipogenesis [37]. Anti-IL-17A mAb has the potential to disrupt the negative feedback loop of IL-17A in the hypothalamus and impact lipogenesis in surrounding adipose tissue, consequently affecting body weight in psoriasis patients.

**Table 4:** Linear mixed model analysis of LCFAs (W8 vs. PSV)

	Measure	Estimate	Std. Err	t value	*P*r ( >|t|)
LCFAs	Anti-IL-17A mAb treatment	-0.198	0.008	-23.971	0.000***
	BMI	0.001	0.001	0.702	0.493
SFAs	Anti-IL-17A mAb treatment	-0.205	0.005	-42.579	0.000***
	BMI	0.001	0.001	0.739	0.472
MUFAs	Anti-IL-17A mAb treatment	0.134	0.041	3.27	0.003**
	BMI	-0.001	0.006	-0.055	0.956
PUFAs	Anti-IL-17A mAb treatment	-0.129	0.026	-4.859	0.000***
	BMI	-0.003	0.005	-0.617	0.544

*Std. Err* Standard error

^**^ Pr ( >|t|) < 0.01

^***^ Pr ( >|t|) < 0.001

The results of serum lipidomics revealed that compared with healthy volunteers, psoriasis patients had higher levels of total LCFAs. However, after the treatment with the anti-IL-17A mAb, there was a substantial decrease in LCFAs. Specifically, SFAs (represented by palmitic acid) and ω-6 PUFAs (represented by arachidonic acid) showed significant increases initially, but these levels decreased considerably after anti-IL-17A mAb treatment. High-fat diets, which are rich in SFAs and ω-6 PUFAs, were found to be consumed more by psoriasis patients than by healthy individuals [38]. Reducing SFA intake through low-fat diets could significantly improve the symptoms of skin changes in psoriasis patients [39]. Abnormally raised SFAs in the samples of psoriasis patients could activate dendritic cells and promote the proliferation of Th17 cells which produce the critical pathogenic factor IL-17A [15, 40]. SFAs act as endogenous ligands for Toll-like receptors, which can activate adipocytes and macrophages in a proinflammatory manner [41]. This activation leads to downstream signal transduction of the NF-kB pathway through TRAF6, IRAK-1, and MyD88, resulting in the production of proinflammatory factors [42–44]. Additionally, SFAs promote keratinocytes to secrete CCL20, which attracts more IL-17A-producing immunologically cells and intensifies skin inflammation [45]. Studies have revealed that ω-6 PUFAs are highly concentrated in healthy skin and are elevated in inflammatory tissue samples [46]. Psoriasis patients exhibit abnormally elevated levels of ω-6 PUFAs in their serum, which can be metabolized to produce proinflammatory bioactive substances with prostanoid-like effects [47]. In addition, the significantly elevated hydroxy fatty acids in the skin and plasma of psoriasis patients are also produced by lipoxygenase metabolizing ω-6 PUFAs in the skin. One of these hydroxy fatty acids, 9-HODE, have been proven to induce the release of inflammatory cytokines and assist in the progress of atherosclerosis [17].

In contrast, the serum of psoriasis patients showed a notable reduction in both MUFAs, such as palmitoleic acid, and ω-3 PUFAs, such as docosahexaenoic acid. Moreover, a significant increase in these LCFAs was observed after anti-IL-17A mAb treatment. Palmitoleic acid has been found to impede neutrophil migration, generate anti-inflammatory factors, and accelerate wound closure [48]. Additionally, both palmitoleic acid and oleic acid process anti-inflammatory properties in endothelial cells, with palmitoleic acid demonstrating a more potent effect than oleic acid [49].

Research has suggested that ω-3 PUFAs have a positive influence against chronic inflammatory disorders, including psoriasis, ulcerative colitis and rheumatoid arthritis, while ω-6 PUFAs can promote inflammation due to the generation of biologically active substances such as prostaglandin E2 [50]. According to the study, psoriasis patients exhibited a metabolic disorder of LCFAs with elevated levels of SFAs and ω-6 PUFAs as well as reduced levels of MUFAs and ω-3 PUFAs in their serum. However, after receiving anti-IL-17A mAb treatment, the LCFA contents in the serum of psoriasis patients approached those of healthy volunteers.

To investigate the correlation between IL-17A and abnormal changes in LCFAs in psoriasis patients, a verification study using IMQ-treated WT and *Tcrd*^*−/−*^ mice was conducted. The results showed a similar trend in the changes in LCFAs in IMQ-treated WT and *Tcrd*^*−/−*^ mice compared to the metabolic changes in LCFAs in psoriasis patients receiving pretherapy and posttreatment with anti-IL-17A mAb. Specifically, the SFA and ω-6 PUFA concentrations rose significantly in IMQ-treated WT mice, with a significant decrease in the MUFA and ω-3 PUFA concentrations. The LCFA contents of *Tcrd*^*−/−*^ mice did not exhibit a significant variation. These findings suggested that anti-IL-17A mAb ameliorated skin lesions in psoriasis patients and regulated their abnormal LCFA metabolism at the same time.

Psoriasis, a systemic skin inflammation, has been found to be associated with cardiovascular diseases (CVDs). Several observational studies have shown that people with psoriasis have a significantly increased chance of CVDs [51]. Previous studies have suggested potential connections between psoriasis and CVD risk factors, indicating the existence of shared underlying mechanisms. Specifically, certain genes involved in fatty acid metabolism have been identified in both psoriasis patients and individuals with cardiovascular risk factors [52]. A serum lipidomic study comparing psoriasis patients and patients with psoriatic comorbidities suggested that SFAs and ω-3 PUFAs increased in psoriasis patients with CVDs. However, there were no significant changes observed in ω-3 PUFAs and MUFAs [18]. Elevated SFAs in human serum have been associated with a higher likelihood of developing CVDs. Specifically, the risks of CVDs and coronary heart disease were found to increase significantly by 50% (95% CI: 1.3-1.71), while the risk of stroke showed a 38% increase (95% CI: 1.05-1.82) [53]. The onset of CVDs has been associated with the excessive intake of ω3 PUFAs and higher ω6/ω3 ratios [54]. Plasma levels of ω-3 PUFAs are associated with lower mortality in CVD patients [55]. A study of Chinese individuals revealed a positive correlation between plasma SFAs and diastolic blood pressure in hypertension patients, while ω-3 PUFAs showed a negative correlation [56]. These studies suggested that there was a similarity in the metabolism of LCFAs between psoriasis patients with CVDs and cardiovascular patients. This study revealed this similarity, specifically increased SFAs and ω-6 PUFAs, in psoriasis patients aligned with the cardiovascular patients or psoriatic patients with CVDs. Disordered LCFAs may be considered as one of the potential factors for cardiovascular risk in psoriasis patients. Furthermore, abnormal LCFAs metabolism is improved in psoriasis patients atfer receiving therapy of anti-IL-17A mAb.

Previous studies have demonstrated abnormal metabolic profiles of LCFAs in psoriasis patients. However, there have been conflicting findings regarding the effects of LCFAs on psoriasis. Although IL-17A plays a significant role in psoriasis, the correlation between abnormal metabolic LCFAs and IL-17A in psoriasis patients has not yet been established. To enhance the reliability of this study on abnormal LCFA metabolism in psoriasis patients, not only were the differences in LCFA metabolism between healthy individuals and psoriasis patients were compared, but also the metabolic changes in LCFAs in each psoriasis patient before and after anti-IL-17A mAb treatment were investigated. Additionally, the serum lipidomic of LCFAs in IMQ-treated WT and *Tcrd*^*−/−*^ mice further supported the correlation between IL-17A and abnormal LCFA metabolism in psoriasis patients. The findings of this study not only explored the correlation between abnormal LCFA metabolism and the key pathogenic factor IL-17A in psoriasis patients but also provided a theoretical foundation for the use of anti-IL-17A mAb in treating the skin lesions of psoriasis patients while improving their abnormal LCFA metabolism.

### Study strengths and limitations

This study has several strengths. First, the researchers established a simple, rapid, and sensitive in-situ extraction derivatization procedure for quantitative LCFAs using the GC–MS analysis platform. Second, the study performed targeted metabolic analysis of LCFA alterations in psoriasis patients receiving pretherapy and posttreatment with anti-IL-17A mAb, which was a unique approach compared to previous metabolic analyses comparing healthy volunteers and psoriasis patients. Finally, to further investigate the correlation between IL-17A and abnormal changes in LCFAs, both IMQ-treated WT and *Tcrd*^*−/−*^ mice were used. While the research provides several innovative insights, it was essential to realize its limitations. The small sample size used in the study may have led to false-positive results. Additionally, the study did not investigate the relationship between lipid metabolism and diet preferences or lifestyle, which could have provided additional context to the findings.

## Conclusion

A rapid and reliable methodology was developed for qualitative and quantitative screening of LCFAs in serum samples. The in situ extraction method enhanced the detection of LCFAs with GC–MS and reduced the analysis time. Serum lipidomics analysis of psoriasis patients receiving pretherapy and posttreatment with anti-IL-17A mAb showed that the treatment ameliorated the skin lesions and regulated the dysregulated metabolism of LCFAs. Alterations in LCFAs of IMQ-treated WT and *Tcrd*^*−/−*^ mice suggest a potential association between the pivotal pathogenic factor IL-17A and the abnormal metabolism of LCFAs among psoriasis patients. Overall, this study provided valuable insights into the effects of serum LCFA metabolism in psoriasis patients treated with anti-IL-17A mAb and emphasized the importance of targeted metabolic profiling in comprehending disease mechanisms.

### Supplementary Information

**Additional file 1:** **Table S1. **Qualitative and quantitative ions of 14 LCFAs in SIM mode. **Table S2. **Concentrations (μM) of LCFAs in serum samples of healthy individuals and psoriasis patients receiving pretherapy and posttreatment with anti-IL-17A mAb. **Table S3. **Concentrations (μM) of LCFAs in serum samples of IMQ-treat WT and *Tcrd*^-/-^ mice. **Fig. S1.** LCFAs derivatization method optimization. To achieve optimal efficiency, 30 μL LCFAs should be heated in the 500 μL 15% BF_3_-CH_3_OH solution (a-c) at 40°C for 30 minutes (d, e). Dichloromethane was also recommended as a superior extraction solvent (f). *P <0.05, **P <0.01, ***P <0.001, ****P <0.0001. **Fig. S2.** Correlation analysis of serum ω-6 (a) and ω-3 (b) PUFAs with PASI scores in psoriasis patients. **Fig. S3. **LCFAs were conducted on ROC curve analysis. AUC values of 10 LCFAs in the PSV/HC (a) and W8/PSV (b) comparisons.

## Data Availability

The data that support the findings of this study are available from the corresponding author upon reasonable request.

## Abbreviations

AUC: Area under the curve
BMI: Body mass index
CVDs: Cardiovascular diseases
FAs: Fatty acids
FAMEs: Fatty acid methyl esters
GC–MS: Gas chromatography–mass spectrometry
HCs: Group of healthy controls
IMQ: Imiquimod
LCFAs: Long-chain fatty acids
IL-17A: Interleukin-17A
LOD: Limit of detection
LOQ: Limit of quantification
mAb: Monoclonal antibody
MUFAs: Monounsaturated fatty acids
PASI: Psoriasis area and severity index
PUFAs: Polyunsaturated fatty acids
PSV: Group of psoriasis patients
SFAs: Saturated fatty acids
ROC: Receiver operating characteristic
RSD: Relative standard deviation
SIM: Selected ion monitor
Vas: Vaseline
WT: Wild-type
W1, W2, W8: Group of psoriasis patients treated with ixekizumab for one, two and eight weeks

## References

[1] Davidson BC, Cantrill RC (1985). Fatty acid nomenclature. A short review. S Afr Med J..

[2] Tvrzicka E, Kremmyda LS, Stankova B, Zak A (2011). Fatty acids as biocompounds: their role in human metabolism, health and disease–a review. Part 1: classification, dietary sources and biological functions. Biomed Pap Med Fac Univ Palacky Olomouc Czech Repub.

[3] Kihara A (2012). Very long-chain fatty acids: elongation, physiology and related disorders. J Biochem.

[4] Serafim V, Tiugan DA, Andreescu N, Mihailescu A, Paul C, Velea I, Puiu M, Niculescu MD (2019). Development and Validation of a LC(-)MS/MS-Based Assay for Quantification of Free and Total Omega 3 and 6 Fatty Acids from Human Plasma. Molecules.

[5] Zhou T, Yang K, Ma Y, Huang J, Fu W, Yan C, Li X, Wang Y (2023). GC/MS-Based analysis of fatty acids and amino acids in H460 cells treated with short-chain and polyunsaturated fatty acids: a highly sensitive approach. Nutrients.

[6] Grzegorczyk EA, Harasim-Symbor E, Lukaszuk B, Harasiuk D, Choromanska B, Mysliwiec P, Zendzian-Piotrowska M, Chabowski A (2018). Lack of pronounced changes in the expression of fatty acid handling proteins in adipose tissue and plasma of morbidly obese humans. Nutr Diabetes.

[7] Patterson AD, Maurhofer O, Beyoglu D, Lanz C, Krausz KW, Pabst T, Gonzalez FJ, Dufour JF, Idle JR (2011). Aberrant lipid metabolism in hepatocellular carcinoma revealed by plasma metabolomics and lipid profiling. Cancer Res.

[8] Chiu HH, Kuo CH (2020). Gas chromatography-mass spectrometry-based analytical strategies for fatty acid analysis in biological samples. J Food Drug Anal.

[9] Griffiths CEM, Armstrong AW, Gudjonsson JE, Barker J (2021). Psoriasis Lancet.

[10] Menter A, Krueger GG, Paek SY, Kivelevitch D, Adamopoulos IE, Langley RG (2021). Interleukin-17 and Interleukin-23: a narrative review of mechanisms of action in psoriasis and associated comorbidities. Dermatol Ther (Heidelb).

[11] Kimball AB, Jemec GBE, Alavi A, Reguiai Z, Gottlieb AB, Bechara FG, Paul C, Giamarellos Bourboulis EJ, Villani AP, Schwinn A (2023). Secukinumab in moderate-to-severe hidradenitis suppurativa (SUNSHINE and SUNRISE): week 16 and week 52 results of two identical, multicentre, randomised, placebo-controlled, double-blind phase 3 trials. Lancet.

[12] Miles EA, Childs CE, Calder PC (2021). Long-Chain Polyunsaturated Fatty Acids (LCPUFAs) and the Developing Immune System: A Narrative Review. Nutrients.

[13] Park YK, Yadav AK, Roshanzadeh A, Ryoo YW, Kim BH, Cha JY, Son YK, Lee NY, Jang BC (2020). 7-MEGA 500 regulates the expression of COX-2, MMP-3 and type 1 procollagen in UVB-irradiated human keratinocytes and dermal fibroblasts. Mol Med Rep.

[14] Qin S, Wen J, Bai XC, Chen TY, Zheng RC, Zhou GB, Ma J, Feng JY, Zhong BL, Li YM (2014). Endogenous n-3 polyunsaturated fatty acids protect against imiquimod-induced psoriasis-like inflammation via the IL-17/IL-23 axis. Mol Med Rep.

[15] Stelzner K, Herbert D, Popkova Y, Lorz A, Schiller J, Gericke M, Kloting N, Bluher M, Franz S, Simon JC, Saalbach A (2016). Free fatty acids sensitize dendritic cells to amplify TH1/TH17-immune responses. Eur J Immunol.

[16] Ikai K (1999). Psoriasis and the arachidonic acid cascade. J Dermatol Sci.

[17] Sorokin AV, Domenichiello AF, Dey AK, Yuan ZX, Goyal A, Rose SM, Playford MP, Ramsden CE, Mehta NN (2018). Bioactive lipid mediator profiles in human psoriasis skin and blood. J Invest Dermatol.

[18] Mysliwiec H, Baran A, Harasim-Symbor E, Mysliwiec P, Milewska AJ, Chabowski A, Flisiak I (2017). Serum fatty acid profile in psoriasis and its comorbidity. Arch Dermatol Res.

[19] Perez-Navarro J, Da Ros A, Masuero D, Izquierdo-Canas PM, Hermosin-Gutierrez I, Gomez-Alonso S, Mattivi F, Vrhovsek U (2019). LC-MS/MS analysis of free fatty acid composition and other lipids in skins and seeds of Vitis vinifera grape cultivars. Food Res Int.

[20] Chen ML, Shah V, Patnaik R, Adams W, Hussain A, Conner D, Mehta M, Malinowski H, Lazor J, Huang SM (2001). Bioavailability and bioequivalence: an FDA regulatory overview. Pharm Res.

[21] Tesfa M, Zewotir T, Derese SA, Belay DB, Shimelis H (2023). Linear mixed model to identify the relationship between grain yield and other yield related traits and genotype selection for sorghum. Heliyon.

[22] MacGee J, Allen KG (1974). Preparation of methyl esters from the saponifiable fatty acids in small biological specimens for gas-liquid chromatographic analysis. J Chromatogr.

[23] Rizzo AM, Montorfano G, Negroni M, Adorni L, Berselli P, Corsetto P, Wahle K, Berra B (2010). A rapid method for determining arachidonic:eicosapentaenoic acid ratios in whole blood lipids: correlation with erythrocyte membrane ratios and validation in a large Italian population of various ages and pathologies. Lipids Health Dis.

[24] Kuiper HC, Wei N, McGunigale SL, Vesper HW (2018). Quantitation of trans-fatty acids in human blood via isotope dilution-gas chromatography-negative chemical ionization-mass spectrometry. J Chromatogr B Analyt Technol Biomed Life Sci.

[25] Kish-Trier E, Schwarz EL, Pasquali M, Yuzyuk T (2016). Quantitation of total fatty acids in plasma and serum by GC-NCI-MS. Clinical Mass Spectrometry.

[26] Tu J, Yin Y, Xu M, Wang R, Zhu ZJ (2017). Absolute quantitative lipidomics reveals lipidome-wide alterations in aging brain. Metabolomics.

[27] Tsoukalas D, Fragoulakis V, Sarandi E, Docea AO, Papakonstaninou E, Tsilimidos G, Anamaterou C, Fragkiadaki P, Aschner M, Tsatsakis A (2019). Targeted metabolomic analysis of serum fatty acids for the prediction of autoimmune diseases. Front Mol Biosci.

[28] Cai Y, Xue F, Fleming C, Yang J, Ding C, Ma Y, Liu M, Zhang HG, Zheng J, Xiong N, Yan J (2014). Differential developmental requirement and peripheral regulation for dermal Vgamma4 and Vgamma6T17 cells in health and inflammation. Nat Commun.

[29] Cai Y, Xue F, Qin H, Chen X, Liu N, Fleming C, Hu X, Zhang HG, Chen F, Zheng J, Yan J (2019). Differential Roles of the mTOR-STAT3 Signaling in Dermal gammadelta T Cell Effector Function in Skin Inflammation. Cell Rep.

[30] Villanueva J, Carrascal M, Abian J (2014). Isotope dilution mass spectrometry for absolute quantification in proteomics: concepts and strategies. J Proteomics.

[31] Furue M, Furue K, Tsuji G, Nakahara T (2020). Interleukin-17A and Keratinocytes in Psoriasis. Int J Mol Sci.

[32] Saraceno R, Schipani C, Mazzotta A, Esposito M, Di Renzo L, De Lorenzo A, Chimenti S (2008). Effect of anti-tumor necrosis factor-alpha therapies on body mass index in patients with psoriasis. Pharmacol Res.

[33] Tan E, Baker C, Foley P (2013). Weight gain and tumour necrosis factor-alpha inhibitors in patients with psoriasis. Australas J Dermatol.

[34] Wu MY, Yu CL, Yang SJ, Chi CC (2020). Change in body weight and body mass index in psoriasis patients receiving biologics: a systematic review and network meta-analysis. J Am Acad Dermatol.

[35] Gerdes S, Pinter A, Papavassilis C, Reinhardt M (2020). Effects of secukinumab on metabolic and liver parameters in plaque psoriasis patients. J Eur Acad Dermatol Venereol.

[36] Wang HN, Huang YH (2020). Changes in metabolic parameters in psoriatic patients treated with secukinumab. Ther Adv Chronic Dis.

[37] Ahmed M, Gaffen SL (2013). IL-17 inhibits adipogenesis in part via C/EBPalpha PPARgamma and Kruppel-like factors. Cytokine.

[38] Zamboni S, Zanetti G, Grosso G, Ambrosio GB, Gozzetti S, Peserico A (1989). Dietary behaviour in psoriatic patients. Acta Derm Venereol Suppl (Stockh).

[39] Rucevic I, Perl A, Barisic-Drusko V, Adam-Perl M (2003). The role of the low energy diet in psoriasis vulgaris treatment. Coll Antropol.

[40] Zhang Y, Li Q, Rao E, Sun Y, Grossmann ME, Morris RJ, Cleary MP, Li B (2015). Epidermal Fatty Acid binding protein promotes skin inflammation induced by high-fat diet. Immunity.

[41] Lee JY, Sohn KH, Rhee SH, Hwang D (2001). Saturated fatty acids, but not unsaturated fatty acids, induce the expression of cyclooxygenase-2 mediated through Toll-like receptor 4. J Biol Chem.

[42] Frommer KW, Schaffler A, Rehart S, Lehr A, Muller-Ladner U, Neumann E (2015). Free fatty acids: potential proinflammatory mediators in rheumatic diseases. Ann Rheum Dis.

[43] Leiguez E, Giannotti KC, Moreira V, Matsubara MH, Gutierrez JM, Lomonte B, Rodriguez JP, Balsinde J, Teixeira C (2014). Critical role of TLR2 and MyD88 for functional response of macrophages to a group IIA-secreted phospholipase A2 from snake venom. PLoS One.

[44] Reynolds CM, McGillicuddy FC, Harford KA, Finucane OM, Mills KH, Roche HM (2012). Dietary saturated fatty acids prime the NLRP3 inflammasome via TLR4 in dendritic cells-implications for diet-induced insulin resistance. Mol Nutr Food Res.

[45] Nakamizo S, Honda T, Adachi A, Nagatake T, Kunisawa J, Kitoh A, Otsuka A, Dainichi T, Nomura T, Ginhoux F (2017). High fat diet exacerbates murine psoriatic dermatitis by increasing the number of IL-17-producing gammadelta T cells. Sci Rep.

[46] Cunnane SC (2003). Problems with essential fatty acids: time for a new paradigm?. Prog Lipid Res.

[47] Lapenna A, Laxton RC, Ye S. Comment on: “A promoter polymorphism (rs17222919, -1316T/G) of ALOX5AP is associated with intracerebral hemorrhage in Korean population” by Hwan Kim D. et al. [Prostaglandins Leukot. Essent. Fatty Acids 85 (2011) 115-120]. Prostaglandins Leukot Essent Fatty Acids 2012, 86:135-136. 10.1016/j.plefa.2012.01.001 22321777

[48] Weimann E, Silva MBB, Murata GM, Bortolon JR, Dermargos A, Curi R, Hatanaka E (2018). Topical anti-inflammatory activity of palmitoleic acid improves wound healing. PLoS One.

[49] de Souza CO, Valenzuela CA, Baker EJ, Miles EA, Rosa Neto JC, Calder PC (2018). Palmitoleic acid has stronger anti-inflammatory potential in human endothelial cells compared to oleic and palmitic acids. Mol Nutr Food Res.

[50] Simopoulos AP (2002). Omega-3 fatty acids in inflammation and autoimmune diseases. J Am Coll Nutr.

[51] Gao N, Kong M, Li X, Zhu X, Wei D, Ni M, Wang Y, Hong Z, Dong A (2022). The association between psoriasis and risk of cardiovascular disease: a mendelian randomization analysis. Front Immunol.

[52] Piaserico S, Orlando G, Messina F (2022). Psoriasis and cardiometabolic diseases: shared genetic and molecular pathways. Int J Mol Sci.

[53] Li Z, Lei H, Jiang H, Fan Y, Shi J, Li C, Chen F, Mi B, Ma M, Lin J, Ma L (2022). Saturated fatty acid biomarkers and risk of cardiometabolic diseases: a meta-analysis of prospective studies. Front Nutr.

[54] Simopoulos AP (2008). The importance of the omega-6/omega-3 fatty acid ratio in cardiovascular disease and other chronic diseases. Exp Biol Med (Maywood).

[55] Rodriguez D, Lavie CJ, Elagizi A, Milani RV (2022). Update on Omega-3 polyunsaturated fatty acids on cardiovascular health. Nutrients.

[56] Huang T, Shou T, Cai N, Wahlqvist ML, Li D (2012). Associations of plasma n-3 polyunsaturated fatty acids with blood pressure and cardiovascular risk factors among Chinese. Int J Food Sci Nutr.

