# Supplementary material for: Serum lipidomic study of long-chain fatty acids in psoriasis patients prior to and after anti-IL-17A monoclonal antibody treatment by quantitative GC‒MS analysis with in situ extraction
Source: Lipids Health Dis. 2024 Jan 8;23:6. doi: 10.1186/s12944-023-01999-6 (PMC10773056; doi:10.1186/s12944-023-01999-6)

**SUPPLEMENTARY INFORMATON**

Serum Lipidomic Study of Long-Chain Fatty Acids in Psoriasis Patients prior to and after anti-IL-17A Monoclonal Antibody Treatment by Quantitative GC‒MS Analysis with In Situ Extraction

XiaoYu Guo^a^, Jianglu Zhou^a^, Hong Yu^b^, Han Cao^c^, Xia Li^c^, Qing Hu^b*^ and YunQiu Yu ^a*^

^a^School of Pharmacy, Fudan University, Shanghai 201203, PR China

^b^NMPA Key Laboratory for Quality, Control of Traditional Chinese Medicine, Shanghai Institute for Food and Drug Control, Shanghai, 201203, PR China.

^c^Department of Dermatology, Ruijin Hospital, School of Medicine, Shanghai Jiaotong University, Shanghai 200025, PR China

*Corresponding author(s): YunQiu Yu, E-mail: yqyu@shmu.edu.cn; Qing Hu, E-mail: huqingyjs@163.com

**Table S1.** Qualitative and quantitative ions of 14 LCFAs in SIM mode

| **LCFA** | **Retention time (min)** | **Qualitative ions (m/z)** | **Qualitative ions of ISO (m/z)** |
| --- | --- | --- | --- |
| 12:0 | 6.706 | 74.0*、214.1 | 76.0*、216.1 |
| 14:0 | 7.986 | 74.0*、242.2 | 76.0*、244.2 |
| 16:1 | 9.516 | 55.0*、268.0 | 59.0*、284.2 |
| 16:0 | 9.709 | 143.0、270.2* | 145.1、272.2* |
| 18:2 | 11.568 | 263.1、294.2* | 264.2、295.2* |
| 18:1 | 11.642 | 264.2*、296.2 | 266.2*、298.2 |
| 18:0 | 11.947 | 143.0、298.2* | 145.1、300.3* |
| 20:4 | 13.636 | 79.0*、150.1 | 83.0*、154.0 |
| 20:5 | 13.718 | 201.1*、215.1 | 206.0*、220.0 |
| 20:0 | 14.567 | 74.0*、326.2 | 76.0*、328.3 |
| 22:6 | 16.243 | 79.0*、105.0 | 85.0*、113.0 |
| 22:0 | 17.435 | 311.2、354.3* | 315.3、358.3* |
| 24:0 | 19.436 | 339.3、382.4* | 343.3、386.4* |
| 26:0 | 20.878 | 367.3、410.4* | 371.3、414.4* |

（* quantitative ions）

**Table S2.** Concentrations (μM) of LCFAs in serum samples of healthy individuals and psoriasis patients receiving pretherapy and posttreatment with anti-IL-17A mAb

| **LCFAs** | **HC** | **PSV** | **W1** | **W2** | **W8** |
| --- | --- | --- | --- | --- | --- |
| 12:0 | 1.17±0.47 | 2.19±0.57*** | 1.63±0.87 | 1.45±0.61^#^ | 1.27±0.56^##^ |
| 14:0 | 30.97±5.76 | 40.21±6.09*** | 36.83±5.54 | 33.42±6.14^#^ | 34.05±6.55 |
| 16:1 | 25.49±6.98 | 18.42±2.84*** | 20.51±2.71 | 22.09±4.22 | 22.14±4.79^#^ |
| 16:0 | 4344.26±553.29 | 5787.41±718.52**** | 5313.94±930.55 | 4911.74±708.12^#^ | 4155.04±792.08^##^ |
| 18:2 | 143.01±38.69 | 238.08±77.04*** | 188.57±47.19 | 177.33±38.04^#^ | 173.93±40.63^##^ |
| 18:1 | 308.15±120.27 | 197.68±53.86* | 264.67±57.61 | 280.43±92.16 | 346.79±213.17^#^ |
| 18:0 | 1296.75±188.35 | 1906.71±235.23**** | 1463.49±225.45^####^ | 1317.68±245.18^####^ | 1245.54±234.63^###^ |
| 20:4 | 13.61±4.93 | 38.02±17.07**** | 28.15±11.23 | 19.96±6.28^#^ | 13.63±3.58^##^ |
| 20:5 | NQ | NQ | NQ | NQ | NQ |
| 20:0 | 5.07±0.52 | 8.13±1.11**** | 7.07±0.99 | 5.41±0.65^###^ | 4.99±0.82^###^ |
| 22:6 | 35.95±18.27 | 15.21±7.13**** | 18.73±6.66 | 20.17±8.12 | 27.61±12.7^#^ |
| 22:0 | NQ | NQ | NQ | NQ | NQ |
| 24:0 | NQ | NQ | NQ | NQ | NQ |
| 26:0 | NQ | NQ | NQ | NQ | NQ |

*There was a significant difference compared with the HC group, **P* <0.05, ***P* <0.01, ****P* <0.001, *****P* <0.0001.

# There was a significant difference compared with the PSV group, ^#^*P* <0.05, ^##^*P* <0.01, ^###^*P* <0.001, ^####^*P* <0.0001.

Values are reported as mean ± SD.

**Table S3.** Concentrations (μM) of LCFAs in serum samples of IMQ-treat WT and *Tcrd^-/-^* mice

| **LCFAs** | ***Tcrd^+/+^*-Vas** | ***Tcrd^-/-^*-Vas** | ***Tcrd^+/+^*-IMQ** | ***Tcrd^-/-^*-IMQ** |
| --- | --- | --- | --- | --- |
| 12:0 | 9.49±3.61 | 7.73±1.93 | 17.88±1.31**** | 6.79±0.72^####^ |
| 14:0 | 21.95±4.21 | 23.56±5.58 | 31.26±2.28** | 20.21±3.52^###^ |
| 16:1 | 16.69±0.71 | 16.83±2.79 | 11.03±0.64**** | 19.55±1.67^####^ |
| 16:0 | 1295.93±163.51 | 1247.13±127.65 | 1619.78±82.94** | 1377.21±111.67^#^ |
| 18:2 | 191.59±25.84 | 184.43±28.11 | 253.14±9.15*** | 151.43±11.05^####^ |
| 18:1 | 185.51±39.97 | 193.34±29.79 | 105.76±15.46*** | 156.76±13.49^#^ |
| 18:0 | 854.18±115.39 | 808.95±214.37 | 1122.51±95.17* | 829.72±144.06^#^ |
| 20:4 | 12.52±1.94 | 11.72±2.17 | 16.74±0.97** | 13.19±1.49^#^ |
| 20:5 | NQ | NQ | NQ | NQ |
| 20:0 | 12.21±2.07 | 11.84±2.99 | 16.12±1.29* | 13.38±1.69 |
| 22:6 | 12.92±0.51 | 13.09±1.82 | 13.61±2.23 | 15.14±1.18 |
| 22:0 | NQ | NQ | NQ | NQ |
| 24:0 | NQ | NQ | NQ | NQ |
| 26:0 | NQ | NQ | NQ | NQ |

*There was a significant difference compared with the *Tcrd^+/+^*-Vas group, **P* <0.05, ***P* <0.01, ****P* <0.001, *****P* <0.0001.

# There was a significant difference compared with the *Tcrd^+/+^*-IMQ group, ^#^*P* <0.05, ^##^*P* <0.01, ^###^*P* <0.001, ^####^*P* <0.0001.

Values are reported as mean ± SD, n=6.

**Fig. S1.** LCFAs derivatization method optimization. To achieve optimal efficiency, 30 μL LCFAs should be heated in the 500 μL 15% BF_3_-CH_3_OH solution (a-c) at 40°C for 30 minutes (d, e). Dichloromethane was also recommended as a superior extraction solvent (f). **P* <0.05, ***P* <0.01, ****P* <0.001, *****P* <0.0001.


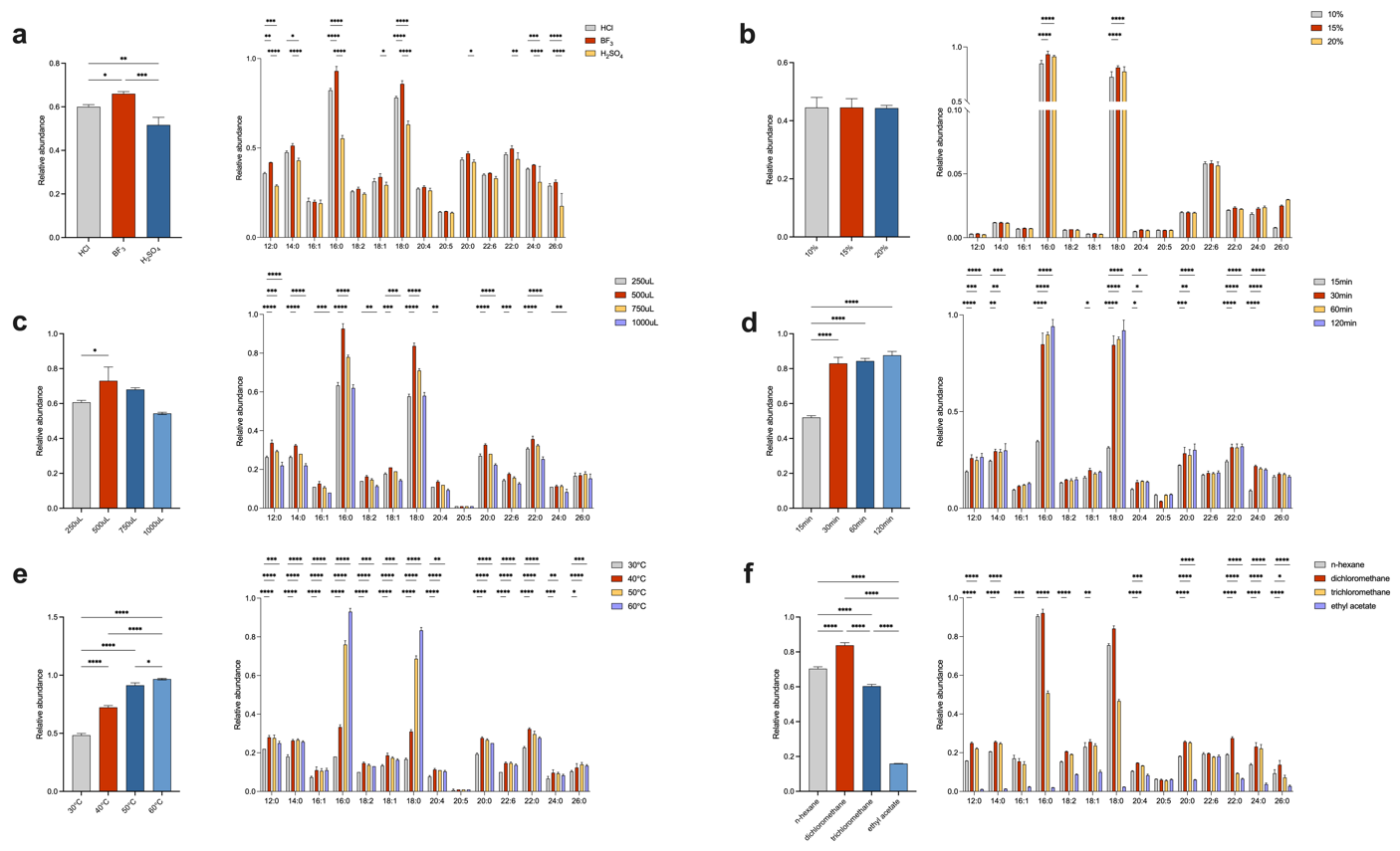


**Fig. S2.** Correlation analysis of serum ω-6 (a) and ω-3 (b) PUFAs with PASI scores in psoriasis patients.


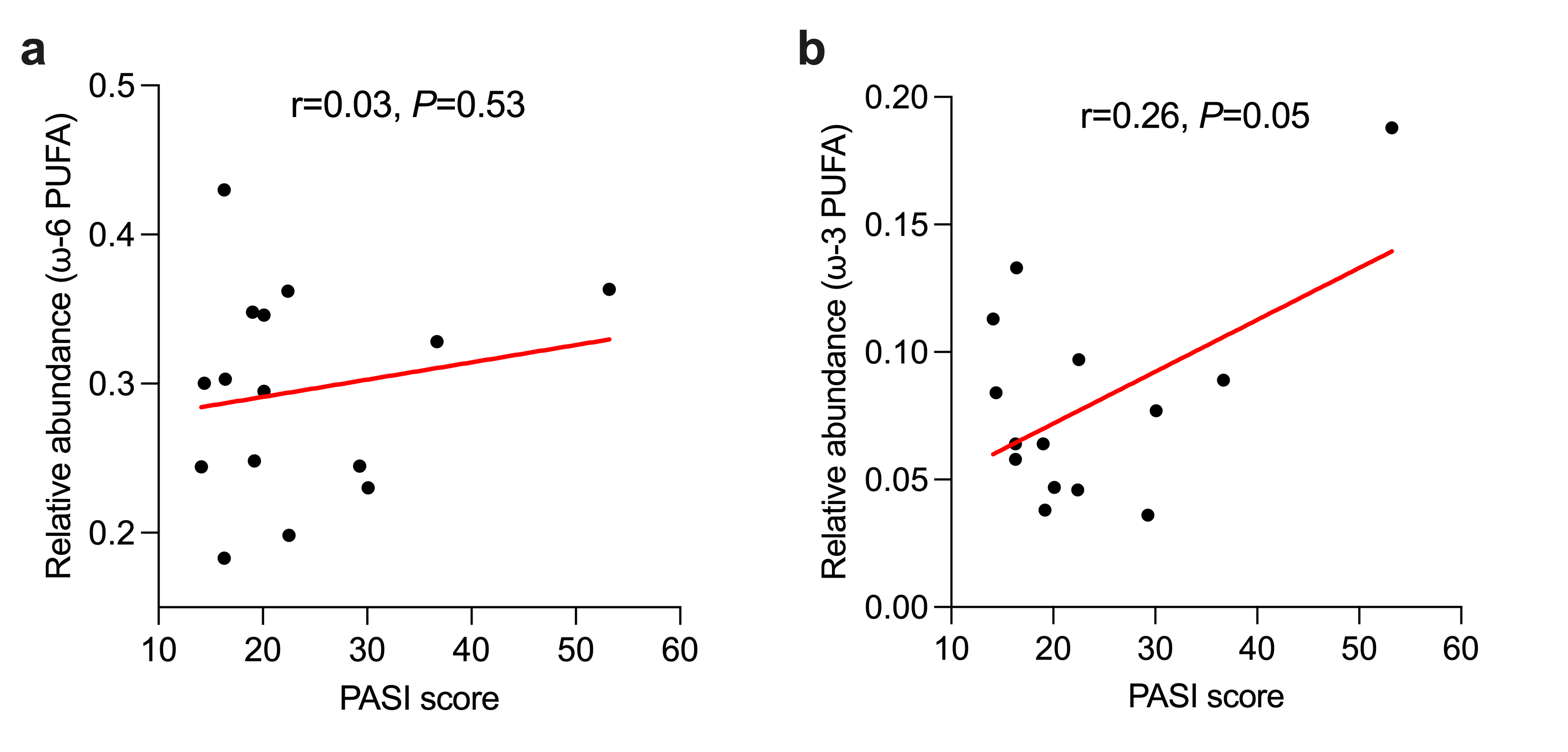


**Fig. S3.** LCFAs were conducted on ROC curve analysis. AUC values of 10 LCFAs in the PSV/HC (a) and W8/PSV (b) comparisons.


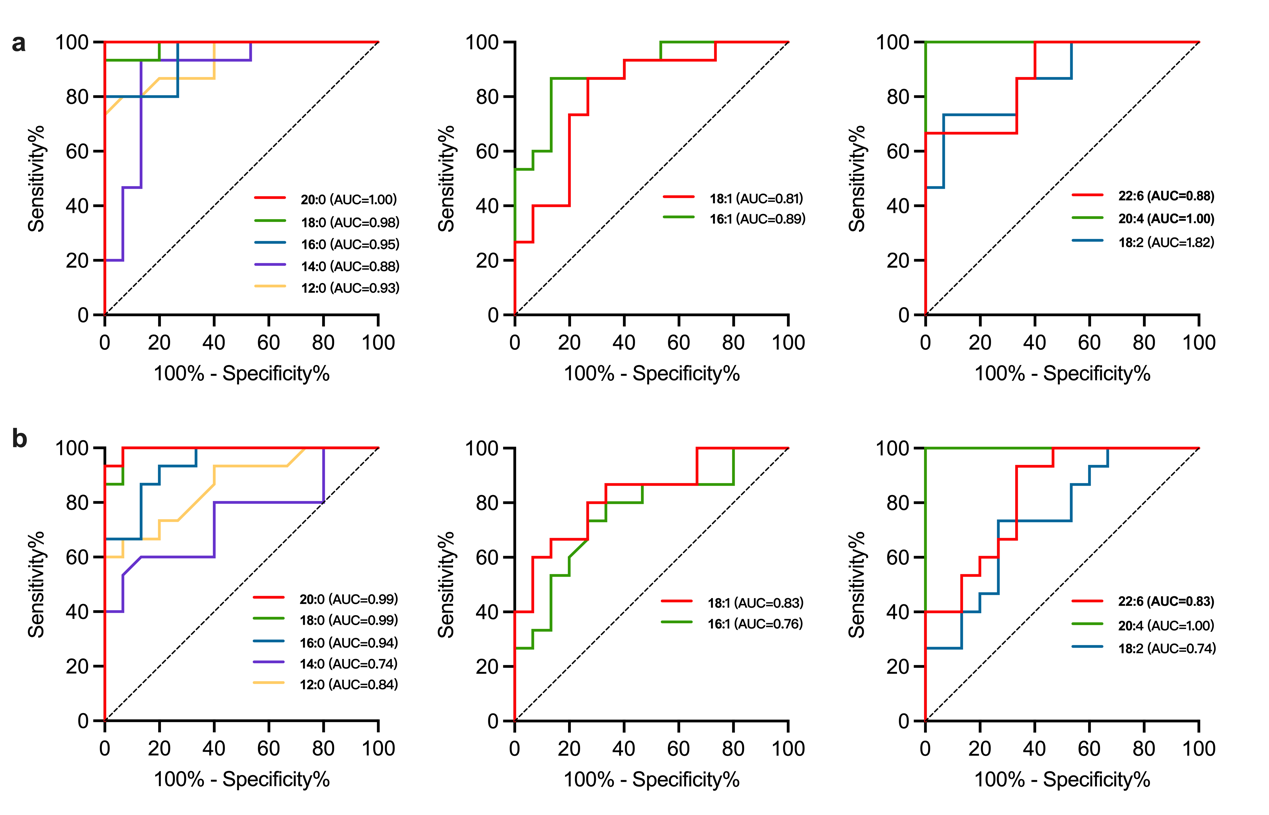

Supplement: Supplementary file 1 — Additional file 1: Table S1. Qualitative and quantitative ions of 14 LCFAs in SIM mode. Table S2. Concentrations (μM) of LCFAs in serum samples of healthy individuals and psoriasis patients receiving pretherapy and posttreatment with anti-IL-17A mAb. Table S3. Concentrations (μM) of LCFAs in serum samples of IMQ-treat WT and Tcrd-/- mice. Fig. S1. LCFAs derivatization method optimization. To achieve optimal efficiency, 30 μL LCFAs should be heated in the 500 μL 15% BF3-CH3OH solution (a-c) at 40°C for 30 minutes (d, e). Dichloromethane was also recommended as a superior extraction solvent (f). *P <0.05, **P <0.01, ***P <0.001, ****P <0.0001. Fig. S2. Correlation analysis of serum ω-6 (a) and ω-3 (b) PUFAs with PASI scores in psoriasis patients. Fig. S3. LCFAs were conducted on ROC curve analysis. AUC values of 10 LCFAs in the PSV/HC (a) and W8/PSV (b) comparisons. [file 12944_2023_1999_MOESM1_ESM.docx]
